# Cross cultural adaptation and validation of a Spanish version of the lower limb functional index

**DOI:** 10.1186/1477-7525-12-75

**Published:** 2014-05-17

**Authors:** Antonio I Cuesta-Vargas, Charles P Gabel, Paul Bennett

**Affiliations:** 1 Departamento de Psiquiatría y Fisioterapia, Facultad de Ciencias de la Salud, Universidad de Malaga, Andalucia Tech, Instituto de Biomedicina de Malaga (IBIMA), Grupo de Clinimetria (AE-14), Málaga, Spain; 2 School of Clinical Science, Faculty of Health at the Queensland University of Technology, Brisbane, Australia; 3 Centre for Healthy Activities, Sport and Exercise of the Faculty of Science at the University of the Sunshine Coast Queensland, Marochydore, Queensland, Australia

**Keywords:** Lower limb, Psychometrics, Outcome assessment, Spanish

## Abstract

**Background:**

The Lower Limb Functional Index (LLFI) is a relatively recently published regional outcome measure. The development article showed the LLFI had robust and valid clinimetric properties with sound psychometric and practical characteristics when compared to the Lower Limb Extremity Scale (LEFS) criterion standard.

**Objective:**

The purpose of this study was cross cultural adaptation and validation of the LLFI Spanish-version (LLFI-Sp) in a Spanish population.

**Methods:**

A two stage observational study was conducted. The LLFI was initially cross-culturally adapted to Spanish through double forward and single backward translation; then subsequently validated for the psychometric characteristics of validity, internal consistency, reliability, error score and factor structure. Participants (n = 136) with various lower limb conditions of >12 weeks duration completed the LLFI-Sp, Western Ontario and McMaster University Osteoarthritis Index (WOMAC) and the *Euroqol Health Questionnaire 5 Dimensions* (EQ-5D-3 L). The full sample was employed to determine internal consistency, concurrent criterion validity, construct validity and factor structure; a subgroup (n = 45) determined reliability at seven days concurrently completing a global rating of change scale.

**Results:**

The LLFI-Sp demonstrated high but not excessive internal consistency (α = 0.91) and high reliability (ICC = 0.96). The factor structure was one-dimensional which supported the construct validity. Criterion validity with the WOMAC was strong (r = 0.77) and with the EQ-5D-3 L fair and inversely correlated (r = -0.62). The study limitations included the lack of longitudinal data and the determination of responsiveness.

**Conclusions:**

The LLFI-Sp supports the findings of the original English version as being a valid lower limb regional outcome measure. It demonstrated similar psychometric properties for internal consistency, validity, reliability, error score and factor structure.

## Introduction

Patient reported outcome (PRO) measures [1,2] are an integral part and process of the management of a patient’s health. These tools are primarily used to objectively determine any response or change in a patient’s status as a consequence of natural healing or the use of an intervention [3]. In this way there is a rapid assimilation and understanding by the clinicians’ and researchers’ of how function and symptoms have changed in response to an intervention on a condition or disease and what effect this has had on the patient’s capabilities [4]. The progression of this patient focused model has seen a gradual shift over the last two decades away from condition or disease specific measures and towards region specific PROs [5,6]. These regional tools reflect a change in status in the three key kinetic-chain regions of the upper and lower [7,8] limbs and the spine [9].

For the kinetic-region of the lower limb, various instruments have been designed to measure and evaluate symptomatology as a single kinetic chain [7,10-15] while others have focused on joints or conditions, particularly for the hip and knee [16-20]. Both regional and condition specific tools are not only used to indicate the effectiveness of an intervention but can also assist in guiding the decision process on what treatments to continue, adopt or change. They also assist in the determination of pre- and post-operative comparison of status and subsequent monitoring of the patient and their recovery during rehabilitation [7]. Generic health related questionnaires such as the *Euroqol Health Questionnaire 5 Dimensions* (EQ-5D-3 L) mix a wide range of quality-of-life dimensions and include a sixth question on overall perceived health-related status. These scales may not provide a complete picture of a particular functional component. Instead the questionnaire’s constructs aim to measure the patient’s overall health status and consequently can serve as a criterion-related validity indicator of general health.

Numerous lower-limb patient reported outcome measures assess function for specific joints [21-25], joint conditions [26-29], or region-specific conditions [30-33]. However, there is limited consensus regarding which tools to use [7,16]. The most commonly advocated and employed lower limb PROs are the regional Lower Extremity Functional Scale (LEFS) [10] and the disease-specific Western Ontario and McMaster Universities (WOMAC) questionnaire [20,34].

Both tools are widely used with translations into several languages. For the LEFS this includes Portuguese, Persian and Dutch, however no validated Spanish version is available. For the WOMAC, a Spanish translated version has been adapted [17] and validated in a chronic population [20]. Despite being developed as a disease specific PRO and shown to have lower clinimetric capacity than the LEFS [34], the WOMAC has served as a regional PRO [34-36]. The WOMAC has multiple translations and by consequence, though not ideal, it is currently the only potentially applicable lower limb regional criterion PRO tool that is validated and available in Spanish [19,20,34] where the psychometric properties were compared to the LEFS [37].

The Lower Limb Functional Index (LLFI) is another recent example of a lower limb regional PRO. The LLFI was published in 2012 and accepted by various professional and governmental organisations [7] as well as independent outcome assessment agencies [38]. The LLFI was developed in line with the WHO-ICF [39] and used a combination of constructs that included body functions, body structures, activities and participation and environmental factors. The LLFI was shown to have strong clinimetric properties [6] that were preferable to those of the LEFS [7]. These properties included the psychometric characteristics of reliability, validity, responsiveness, error measurement and internal consistency. From the practical characteristics perspective [7] it showed brevity, ready transferability to a 100-point scale, ease and rapidity of completion and scoring, low missing responses and suitable readability. The LLFI was also shown [7] to have a single factor structure [36]. In view of the findings of preferable clinimetric properties for this lower limb PRO [7], translation to a Spanish version was warranted. This was supported by findings that comparable PROs in the functional index series for the upper limb [40] and spine [9] were found to be preferable to other advocated English criterion PROs within their development studies and in independent research including translated versions in French and Spanish [41].

A Spanish version of the LLFI had not been adapted and validated to date. This is significant given that Spanish is one of the five most spoken languages and the second widest geographically spoken [42]. Consequently a Spanish version, the LLFI-Sp, was developed to meet this need. Therefore the aims of this paper were: to describe the process of translation and cross-cultural adaptation of the original LLFI to Spanish; and to subsequently assess the five critical psychometric properties of reliability, one-dimensional factor structure, internal consistency, measurement error and concurrent criterion validity for clinical use with Spanish speakers. The hypothesis of the present study is that new LLFI-Sp must show a strong relationship with WOMAC as the region specific criterion.

## Materials and methods

### Design

This was a two stage observational study. Stage 1 involved the initial Spanish translation and cross-cultural adaptation of the LLFI [7]. Stage 2 involved a prospective evaluation of the LLFI-Sp’s critical psychometric properties. This included the concurrent completion, in a Spanish physical therapy outpatients’ population, of the LLFI-Sp with a lower limb regional criterion, the WOMAC [19], and a general health questionnaire, the EQ-5D-3 L [43]. The WOMAC provided a criterion specific comparison for the lower limb while the EQ-5D-3 L enabled a clarification and criterion standard for comparison of the participants’ health status. Two assessors performed the initial and subsequent assessment of data obtained from the questionnaires that were all completed independently by the participants. The assessors were blinded to baseline scores in order to ensure independent collection of outcome data.

### Stage 1 - Translation of the LLFI to the “LLFI-Sp”

**Figure 1:**
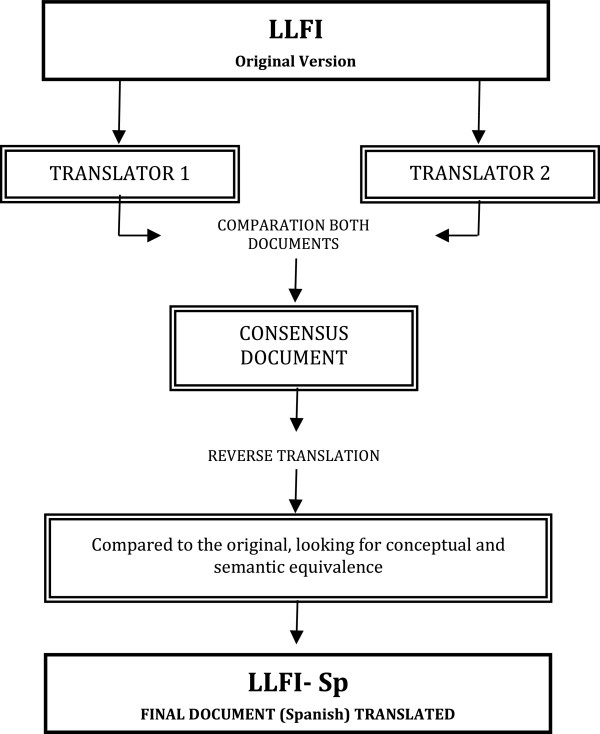
Flowchart of the translation of the Lower Limb Functional Index (LLFI) from English to Spanish.

The primary objective of this aspect of the study was to ensure that the conceptual equivalence of the used terms was retained in a culturally Spanish-specific translation. A direct and reverse translation methodology was applied that involved two native English speakers for the direct translation and one native Spanish speaker for reverse translation. All were specialists in their field, as detailed and recommended in the specialized scientific literature (Figure 1) [44], and as performed in the cross-cultural adaptation to Spanish of other PRO tools [41]. The documents from Translators 1 and 2 showed minimally discernible differences that enabled the final consensus document to be rapidly achieved through a consensus approach of a unanimous agreement on the final version of each item between all translators (Figure 1).

### Stage 2 - Prospective Psychometric Investigation

#### Participants, setting and procedure

A total of 136 consecutive volunteers (48 ± 19 years, 54.4% female) with a variety of chronic (>12 weeks duration) lower limb conditions were recruited from the Physical Therapy Outpatients Clinic at the Malaga University in Spain prior to treatment commencement. The criteria for inclusion were a lower limb injury and diagnosis by a medical practitioner. The chronic status was determined as >12 weeks duration [45] and that any acute treatment had been finalized when the participant was recruited. The chronic status was required as the WOMAC Spanish version had only been validated in chronic patients [20]. This also provided the additional validation of the LLFI in a chronic population as the original study involved an acute population. The presenting conditions and diagnoses were broadly classified into six regional sub-categories (Table 1). The exclusion criteria were age <18 years and poor Spanish language comprehension as required for the completion of the questionnaires and assessed subjectively by the therapist in conjunction with the participant’s feedback on comprehension ability. All participants with eligible criteria completed all three Spanish language self-administered questionnaires, the LLFI-Sp, WOMAC and the EQ-5D-3 L, independently at baseline. Those involved in the reliability study provided a repeat score at day seven and completion of a 5-point global rating of change scale [46]. This study was conducted in accordance with the ethical principles for medical research involving human subjects and approved in January 2011 by the ethics committee of the University of Malaga, (Spain).

**Table 1:** Demographic characteristics and frequency of diagnosis of the study population

***Characteristic***	***Cases (%)***	***Age (years) Mean (sd)***
***Study population***	136	48 ± 19
**Male**	62 (45.6%)	50 ± 20
**Female**	74 (54.4%)	46 ± 25
***Subregion***		
**Hip**	34	56 ± 19
- Bursa	23	
- Capsule	3	
- Impingement	5	
- Other	3	
**Thigh**	8	38 ± 21
- Muscle strain	7	
- Haematoma	1	
**Knee**	58	50 ± 19
- Ligament (MCL, LCL, ACL)	33	
- PFJ	22	
- Other	3	
**Calf and Shin**	9	32 ± 23
- Muscle strain	7	
- Haematoma	2	
**Ankle**	12	46 ± 19
- Ligament	10	
- Other	2	
**Foot**	6	50 ± 21
- Joint	4	
- Other	2	
**Other**	9	59 ± 21
- **Whole leg,**	6	
- **Ulcers,**	1	
- Other	2	

The LLFI is a 25-item regional PRO with 3-point response options of 'Yes’ (points = 1), 'Partly’ or 'Half’ (points = 1/2) and No’ (points = 0) with a raw score range of 0 to 25 points. It requires approximately two minutes to be completed. The score is calculated by simple addition of the responses then multiplied by four for conversion to a percentage scale or maximal loss of function. The total score subtracted from 100 gives a functional score as a percentage of pre-injury or normal status [7].

The WOMAC is a 24-item disease-specific questionnaire developed for patients with hip or knee osteoarthritis (OA). It requires approximately five minutes to be completed. It is a multidimensional scale grouped into three dimensions: pain (five items), stiffness (two items) and physical function (17 items). The version with five response levels for each item was used, scored from 0 to 4, which represented different degrees of intensity. The final score in the Spanish version was validated as determined by adding the aggregate of the three dimensions scores [19,20]. The higher the score the worse the patient’s condition with improvement indicated by an overall score reduction [19,20]. The data were standardized to a 100 percent scale where 0 represented optimal health and 100 the worst possible status. The original and Spanish questionnaires are both previously determined to be reliable, valid and sensitive to the changes in the health status of patients with hip or knee OA [18-20].

The EQ-5D-3 L is a widely used six-item non-disease-specific questionnaire. It has five 3-point response options for different quality-of-life dimensions and a sixth question on overall perceived health-related status Visual Analogue Scale (VAS). The EQ-5D-3 L-VAS is used to reflect the respondent’s self-rated health status on a 100 mm scale and ranked from 'Best Imaginable’ (100) to Worst Imaginable’ (0). The EQ-5D-3 L has been demonstrated as valid and reliable in the Spanish population [42].

*Reliability* was performed using the Intraclass Correlation Coefficient Type 2,1 (ICC_2.1_) test-retest methodology in a randomly selected subgroup of the full sample. Randomization was performed by a computer generated list of numbers that was used against the patient participant number with no refusal or drop outs within in this reliability subgroup (n = 45, 49 ± 3 years, 56.1% female). Their presenting conditions were verified as representative of the six area sub-categories of the full sample. The LLFI-Sp baseline and the repeated measures, taken at seven days [47] following a period during which there was no treatment, were both expressed with a 95% CI. To clarify that no change in status had occurred between the two measurement intervals a 5- point global rating of change was employed with a limitation of one scale point difference [46].

### Statistics

*Descriptive analyses* were applied to calculate means and standard deviations of the demographic variables (Table 1). *Distribution and normality* were determined by the one-sample Kolmogorov-Smirnov tests. *Construct validity* was determined *through* the use of factor structure where a single factor structure would indicate that all items were reflective of the construct of interest – lower limb functional status. Both *construct validity and factor structure* were determined from maximum likelihood extraction (MLE) with the *a-priori* extraction requirements being satisfaction of three critera: screeplot inflection, Eigenvalue >1.0 and variance >10%. We satisfied the recommended minimum ratio of five participants-per-item [48]. *Exploratory factor analysis* indicated a single factor structure was likely therefore more than 100 participants were required. The *internal consistency* was determined from Cronbach’s α coefficient as calculated at an anticipated value range of 0.80-0.95 [49]. A student T-test was developed to check that items behaved the same way for males and females.

**Figure 2:**
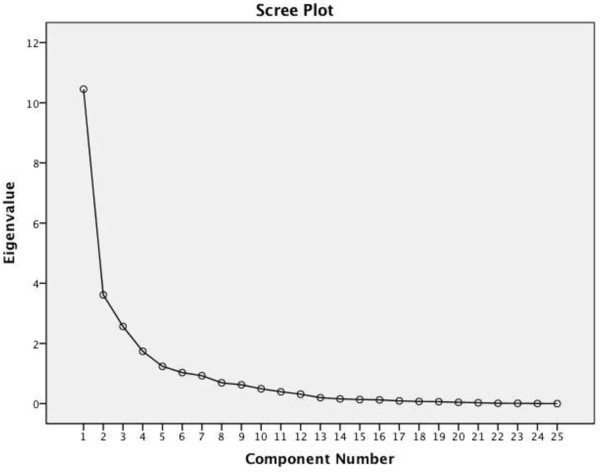
Blant-Altman plot for the test-retest reliability.

The *sensitivity or measurement error score* was determined from the *MDC*_*90*_ analysis that was performed as described by Stratford [50]. The standard error of the measurement (SEM) was calculated using the formula: SEM = s√(1–r), where s = standard deviation of the test scores (SD) of time 1 and time 2, r = the reliability coefficient for the test and Pearson’s correlation coefficient between test and retest values [51]. To investigate statistical agreement between the test scores of Time 1 and time 2 a Bland-Altman test was also performed (Figure 2).

$$\begin{array}{l} \;\mathrm{Thereafter}\;\mathrm{the}\;{\mathrm{MDC}}_{90}\mathrm{was}\;\mathrm{calculated}\;\mathrm{using}\;\mathrm{the}\;\mathrm{formula}:\\ {\mathrm{MDC}}_{90}=\mathrm{SEM}\times \surd 2\times 1.65. \end{array}$$

*Criterion validity* was determined through the concurrent use of the WOMAC for specific criterion validity with the LLFI-Sp measure and with the EQ-5D-3 L total score and EQ-5D-3 L-VAS scores for criterion validity with general health status. The Pearson’s r correlation coefficient used the criteria of poor (r < 0.49), fair (r = 0.50-0.74) and strong (r > 0.75) [52].

The minimum *sample sizes* for the validation study were calculated from the original study where respectively criterion validity was determined through use of Meng’s test of significance and solving for n [53] and reliability was determined from an 80% likelihood of detecting differences between the baseline and repeated measurements. Both calculations allowed for a 15% attrition with p < 0.05 [44]. Power calculations indicated the need for a minimum sample of n ≥ 110 for concurrent criterion validity and n ≥ 45 for reliability [52].

All *statistical analyses* were conducted using the Statistical Package for Social Science version 17.0 (SPSS 17.0) for Windows and LISREL 8.80 [54].

## Results

The demographic and frequency of diagnosis of the sample are detailed in Table 1. The LLFI was translated and back translated with consideration of the Spanish cultural linguistic adaptation to provide the new LLFI-Sp questionnaire without language difficulties or other conceptual misunderstanding (Additional file 1). The mean and standard deviation for LLFI-Sp score were determined (5.88 ± 5.6 points), there were no missing responses and a high degree of *internal consistency* (α = 0.91) was demonstrated with an item range of 0.88 to 0.95.

The *test-retest reliability* was high at (ICC = 0.96) with a range of 0.93 to 0.97. *Measurement error* from SEM and MDC_90_ were respectively 3.12% and 7.12%. No significance gender differences were found in the item responses. The Bland-Altman plot showed a high level of agreement between the test scores at Time 1 and 2 (Figure 2).

**Figure 3:**
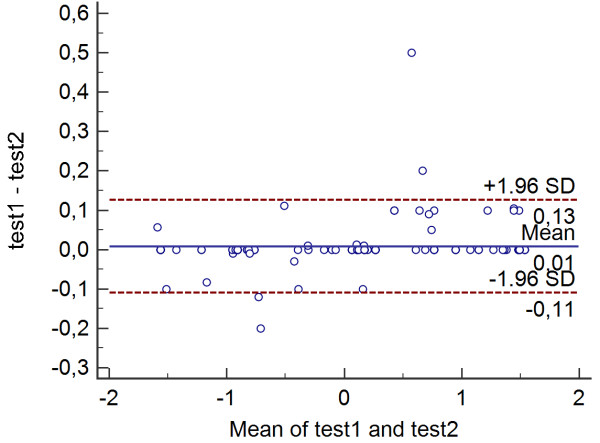
Scree plot of the exploratory one-factor solution.

For *factor analysis* the correlation matrix for the LLFI-Sp was determined as suitable from the Kaiser-Meyer-Oklin values (0.86) and Barlett’s Test of Sphericity (p < 0.001). This indicated that the correlation matrix was unlikely to be an identity matrix and was therefore suitable for MLE. The a-priori requirements for one-dimensional factor structure were verified. The screeplot (see Figure 3) indicated a one-factor solution when all three *a-priori* factors were accounted for. The factor analysis revealed a satisfactory percentage of total variance explained by the one factor at 22.8%. It was noted that eight factors had Eigenvalues >1.0 and accounted for 65.4% of variance; however those with an Eigenvalue >1.0 each accounted for <10% of variance and were shown to be after the screeplot initial inflection point (Figure 3) and consequently not extracted. The item loading for the one-factor solution for the MLE method and average score for each item is shown in Table 2.

**Table 2:** Factor loading items for the one-factor solution, average score and discrimination indices of items (n = 136)

**Question**	**Item**	**Factor loading**	**Item average score**	**Item discr. indices**
1	Stay at home most of time	.780	.25	.259^*^
2	Change positions frequently	.703	.38	.811^**^
3	Avoid heavy jobs	.466	.75	.561^**^
4	Rest more often	.691	.75	.388^*^
5	Get others to do things	.726	.38	.656^**^
6	Pain almost all the time	.690	.38	.822^**^
7	Lifting and carrying	.735	.75	.561^*^
8	Appetite affected	.740	.13	.334^*^
9	Walking/normal recreation/sport	.796	.75	.561^**^
10	Home/family duties and chores	.514	.38	.665^**^
11	Sleep less well	.607	.38	.622^**^
12	Assistance with personal care, hygiene	.706	.13	.690^**^
13	Regular daily activity work/social	.601	.63	.811^**^
14	More irritable/bad tempered	.497	.13	.776^**^
15	Feel weaker or stiffer	.838	.25	.622^**^
16	Transport independence	.687	.13	.863^**^
17	Difficulty or need with dressing (e.g. trousers/pants/shoes and socks)	.849	.25	.480^*^
18	Difficulty changing directions, twisting or turning.	.429	.25	.509^*^
19	Unable to move as fast as I would wish.	.664	.50	.209^*^
20	I have difficulty with prolonged or extended standing.	.658	.50	.863^**^
21	Difficulty bending, squatting and/or reaching down.	.645	.63	.767^**^
22	Difficulty with long or extended walks.	.249	.63	.523^*^
23	Difficulty with steps and stairs.	.471	.88	.624^**^
24	Difficulty with sitting for prolonged or extended times.	.620	.25	.782^**^
25	Problems with my balance on uneven surfaces and / or with unaccustomed footwear.	.394	.63	.803^**^

*p < 0.05.

**p < 0.01.

*Criterion validity* determined from the relationship between the LLFI-Sp and WOMAC was strong (r = 0.77) but fair and inversely related for the LLFI-Sp and the EQ-5D-3 L (r = -0.62) and EQ-5D-3 L-VAS (r = -0.58).

## Discussion

### Main findings

The LLFI was translated to provide a cross-cultural adaptation to the Spanish language. The translation process ensured the conceptual equivalence of the used terms. This provided accessibility to the LLFI for the second largest geographically used language. The psychometric properties, specifically construct and criterion validity, reliability and internal consistency were determined and found to be strong and the single factor structure indicated a single summated score could be used [36].

The cross-cultural adaptation of the LLFI into Spanish enables clinicians in Spanish speaking settings to compare outcomes following their treatments and interventions affecting the lower limb. The procedure of cross-cultural adaptation of a scale has been used in previous studies for different scales to be applied in the Spanish context [43,44]. It is critical to employ research measures that are valid and reliable but they must also be both culturally and linguistically appropriate [41].

The one-factor solution that emerged in the factor analysis accounted for a significant proportion of variance. Though this value is lower than the 30.3% found in the original study it is still an acceptable level for a 25-item questionnaire [52]. This evidence also supports the presence of construct validity. A one-factor solution is critical if a PRO is to be used with a single summated score [36], and subsequently reflect the construct for which it is primary used [6] – that of representation of the functional status of the lower limb as a single kinetic chain [7].

Four further critical psychometric properties of the LLFI-Sp were shown to be well supported. The internal consistency analysis at α = 0.91 was identical to that of the original English version [7], which sits below the accepted 0.95 thresholds for item redundancy [36]. The test-retest reliability or reproducibility (r = 0.96 and Figure 2) was also equivalent to the original instrument (0.97) [7]. The criterion validity with the WOMAC was demonstrated as strong (r = 0.77) suggesting transferability and substitution may be an option. This is supported by the remaining psychometric factors that are preferable and that the LLFI was designed as a lower limb regional tool rather than a joint or condition specific measure, as was the case for the WOMAC. The EQ-5D-3 L was fair and inversely correlated (r = -0.62) but at a lower level suggesting substitution for general health measurement and vice versa is unlikely to be appropriate. The directional trend however supports the construct validity and the determination of the LLFI-Sp as appropriate in terms of face and content validity as a deteriorating health correlates to worsening function. The level of measurement error with an MDC_90_ of 7.12% was comparable that larger than that of the original LLFI at 6.6%.

### Study strengths and limitations

The *strengths of the study* include the prospective nature and the adequate number of subjects that provided a suitable sample size and power of analysis. The inclusion of consecutive patients, independence of the assessors and referral source as well as the broad diagnosis and category representations suggests limited selection bias and the potential for population generalizability [48]. The results for the psychometric properties support the findings of the previous research on the original English version of the LLFI indicating broad cross-cultural adaptions would be appropriate to other diverse socioeconomic, cultural and linguistic groups. The LLFI-Sp also provides a means of comparing lower limb health status in Spanish-speaking patients with their English-speaking counterparts in countries with a high Spanish-speaking population such as the United States.

The *study limitations* include the lack of longitudinal data regarding other psychometric properties, including responsiveness or sensitivity to change, and a minimal clinically important difference. Also face and content validity which should also be performed in a translated instrument were not determined within a patient focus subgroup but from the transilational aspects only. The translation process ensured the conceptual equivalence of the used terms, however only the translators and no with cognitive interviews tested this before validation. The determination of construct validity through the use of factor analysis represents only one possible statistical method of testing this property. A construct is not restricted to one set of observable indicators or attributes and additional indicators will require consideration in future research. Similarly, the practical characteristics were not determined. The results are applicable only to the Spanish speaking population from Spain. The inclusion of Hispanic/Latino/South American participants in future studies could potentially provide confirming or conflicting linguistic information due to the cultural and ethnic difference with respect to the Spanish participants and their cultural diversity in terms of European versus the Americas, North, Central and South.

## Conclusions

The LLFI is translated and cross-culturally adapted to Spanish for the first time. The psychometric properties of this LLFI Spanish version are also reported with the determined values found to be satisfactory and supportive of the findings of the LLFI scale in the English format, particularly in the areas of internal consistency, reliability, factor structure and error score. Consequently the LLFI-Sp may be useful in Spanish-speaking populations and for making cross-ethnic and cross-cultural comparisons in other English speaking countries with a high Spanish-speaking population.

## Competing interest

The authors declare that they have no competing interests.

## Authors’ contributions

All the authors have made contributions to conception of this study. AIC-V and PCG participated in the analysis and interpretation of data and were involved in drafting the manuscript or revising it critically for important intellectual content. All the authors have given final approval of the version to be published.

## Supplementary Material

Additional file 1 The Spanish version of the Lower Limb functional index. Click here for file
