# Supplementary material for: Cross cultural adaptation and validation of a Spanish version of the lower limb functional index
Source: Health Qual Life Outcomes. 2014 May 17;12:75. doi: 10.1186/1477-7525-12-75 (PMC4038853; doi:10.1186/1477-7525-12-75)
Supplement: Additional file 1 — The Spanish version of the Lower Limb functional index. [file 1477-7525-12-75-S1.docx]

**Additional file 1: The Spanish Version of the LLFI**

**LOWER LIMB FUNCTIONAL INDEX (Spanish versión) FECHA:**

**NOMBRE: LESIÓN**

**PIERNA IZQUIERDA**  **PIERNA DERECHA**

**POR FAVOR, COMPLETE:** Sus piernas pueden hacer que sea difícil hacer algunas cosas que hace normalmente. Esta lista contiene frases que la gente usa para describir esos problemas en ellos mismos. Piense en usted en los últimos días. **Si una frase lo describe, marque esa casilla. Si no es así, déjela en blanco. Si una frase sólo le describe parcialmente, marque la mitad de la casilla.**

**SOBRE MI/S PIERNA/S:**

1 Me quedo en casa la mayor parte del tiempo.

2 Cambio frecuentemente de postura para aliviar el dolor.

3 Evito hacer trabajos pesados (ejemplo: limpiar, levantar más de 5kg, trabajar el jardín, etc).

4. Paro a descansar más a menudo.

5. Pido a los demás que hagan las cosas por mi.

6. Tengo dolor/problema casi todo el tiempo.

7. Tengo dificultad para levantar y cargar peso (ejemplo: bolsas, compras de hasta 5 kg., etc.).

8. Mi apetito es diferente.

9. El caminar o el hacer mis actividades deportivas y recreativas están afectadas.

10. Tengo dificultad con las tareas normales de la casa y la familia.

11. Duermo peor.

12. Necesito ayuda con mi cuidado personal (ejemplo: la ducha y la higiene).

13. Mis actividades normales diarias (trabajo, actividades sociales) están afectadas.

14. Estoy más irritable y/o de peor humor.

15. Me siento débil y/o rígido.

16. Mi independencia en el transporte está afectada (conducir/transporte público).

17. Tengo dificultad o necesito ayuda para vestirme (ejemplo: pantalones/zapatos/calcetines).

18. Tengo dificultad para cambiar de dirección, torcer y girar.

19. Soy incapaz de moverme tan rápido como desearía.

20. Tengo dificultad para permanecer mucho tiempo de pie.

21. Tengo dificultad para agacharme, ponerme en cuclillas.

22. Tengo dificultad con los paseos prolongados.

23. Tengo problemas con los escalones y las escaleras.

24. Tengo problemas para estar sentado/a durante un tiempo.

25. Tengo problemas con el equilibrio en superficies irregulares o con el calzado especial

**LLFI PUNTUACIÓN: Para puntuar la parte superior sumar las cajas marcadas:**

**TOTAL (LLFI puntos) 100 Escala: 100 – (TOTALx4) = %**

**MDC (90% CI):** 6.67% o 1.67 LLFI-puntos. Una puntuación menor a ésta puede ser debido a error

Spanish translation courtesy of Dr. Cuesta-Vargas, Faculty of Health Sciences, University of Malaga, Spain
